# The use of topical ruxolitinib 1.5% cream in frontal fibrosing alopecia: A case report

**DOI:** 10.1016/j.jdcr.2024.04.034

**Published:** 2024-05-04

**Authors:** Deesha Desai, Ambika Nohria, Kristen Lo Sicco, Jerry Shapiro

**Affiliations:** a University of Pittsburgh School of Medicine, Pittsburgh, Pennsylvania; b The Ronald O. Perelman Department of Dermatology, NYU Grossman School of Medicine, New York, New York

**Keywords:** frontal fibrosing alopecia, JAK inhibitor, ruxolitinib

## Introduction

Topical ruxolitinib 1.5% cream serves as a selective Janus kinase (JAK) inhibitor designed to target JAK1 and JAK2 enzymes, both recognized contributors to inflammation in the skin and scalp.^1^ Presently, this treatment has received Food and Drug Administration approval for addressing mild to moderate atopic dermatitis and nonsegmental vitiligo in adults and adolescents aged 12 years and older, whose conditions are not effectively managed by alternative treatments.^2^, ^3^, ^4^ However, its potential as a therapeutic modality in diverse dermatologic conditions, including frontal fibrosing alopecia (FFA), remains largely unexplored in clinical practice with limited evidence in scientific literature primarily concentrating on the utilization of topical tofacitinib.^5^^,^^6^ Additionally, the current literature predominantly emphasizes the potential utilization of oral JAK inhibitors in scarring alopecia rather than exploring its topical formulation. However, topical ruxolitinib 1.5% cream may present a more favorable safety profile, higher tolerability, and fewer concerns regarding side effects compared to oral JAK inhibitors. Herein, we present a unique case where topical ruxolitinib 1.5% cream demonstrated efficacy in stabilizing the frontal hairline, alleviating physical symptoms, and clearing facial papules in a patient with FFA who was previously unresponsive to conventional therapies.

## Report of a case

**Fig 1:**
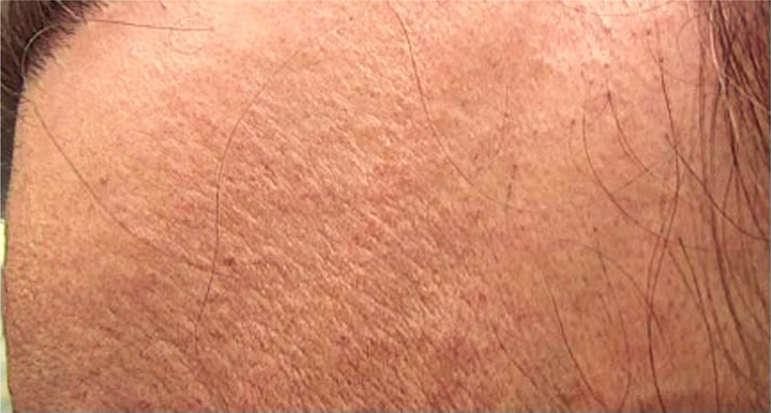
Facial papules prior to topical ruxolitinib 1.5% cream.

**Fig 2:**
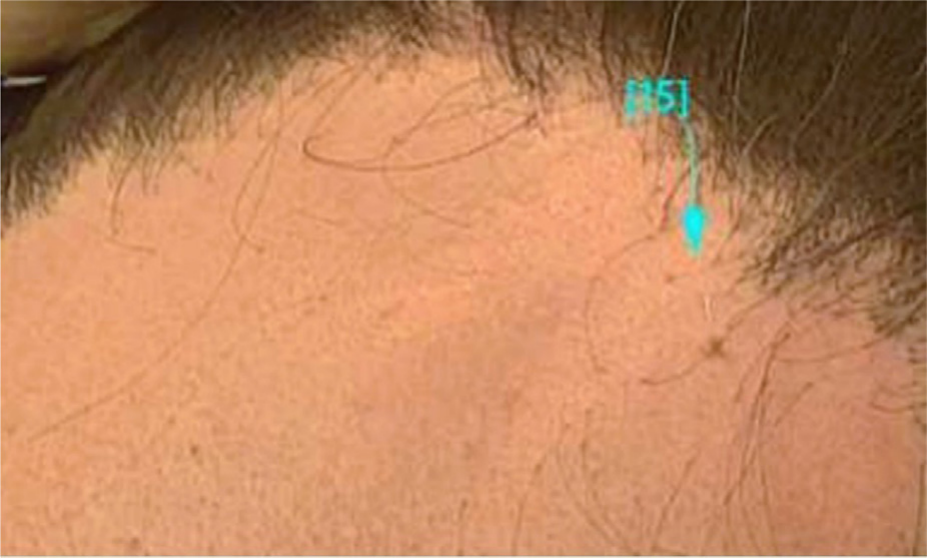
Facial papules after topical ruxolitinib 1.5% cream.

A 55-year-old male with a medical history including alopecia areata presented to the dermatology clinic due to concerns about rapid recession of the frontal hairline accompanied by scalp and eyebrow tenderness, pruritus, and the presence of facial papules. The patient's initial encounter with hair loss occurred 10 years prior, presenting as alopecic patches in the beard and scalp, clinically consistent with alopecia areata, which were effectively managed with intralesional triamcinolone injections at the time and have remained quiescent since. However, 4 years ago, there was a sudden regression of the frontal hairline, which progressed in prominence over time. Scalp and eyebrow pruritus, tenderness, and facial papules were also noted. On physical exam, bilateral temporal recession and follicular dropout were observed, with hairline measurements quantified at 13 cm from the right outer canthus and 12 cm from the left outer canthus. The initial treatment plan included use of doxycycline hyclate 100 mg twice a day followed by hydroxychloroquine 200 mg twice a day as antilymphocytic agents, intralesional triamcinolone injections, tacrolimus 0.3% mixed with Cetaphil cleaner, topical clobetasol 0.05% solution, and pioglitazone 15 mg/d as antiinflammatory agents, and topical minoxidil 5% solution, oral minoxidil 5 mg/d, and dutasteride 0.5 mg/d as simultaneous agents to enhance overall hair density along with excimer narrow band UV-B laser as an adjunctive therapy. While the patient initially reported mild improvement in shedding, facial papules, and physical symptoms such as itching, burning, and pain, a recurrence of these issues was noted 5 months after initiating therapy. Despite modifications to the medication regimen, symptoms persisted. However, 2 years into treatment, topical ruxolitinib 1.5% cream was introduced in addition to the previously unsuccessful treatment regimen. Initially, when the patient used topical ruxolitinib 1.5% cream sparingly, there was no improvement in symptoms. Thereafter, the patient applied topical ruxolitinib 1.5% cream in more generous amounts once daily along the frontal hairline, using 2 tubes per month. He reported that topical ruxolitinib 1.5% cream successfully cleared facial papules [Figs 1 and 2] and led to resolution of physical symptoms after 3 months of use, as well as stabilization of the frontal hairline which was confirmed by clinical evaluation and trichoscopy.

## Discussion

Topical ruxolitinib 1.5% cream currently stands as the sole Food and Drug Administration-approved topical JAK inhibitor formulation in the United States, having received approval in September 2021 for atopic dermatitis followed by approval for vitiligo in July 2022.^2^, ^3^, ^4^ This medication primarily functions by inhibiting the Janus kinase - signal transducers and activators of transcription pathway, which mediates the signaling of various cytokines and growth factors involved in pro-inflammatory pathways.^1^

The pathomechanism of FFA is believed to involve an inflammatory etiology, with a lymphocytic infiltrate targeting the follicular bulge, causing irreversible damage to epithelial stem cells.^7^ Furthermore, a recent study has explored the possible indication that FFA may be triggered by antigenic stimuli originating in the melanocytes of the upper hair follicle, particularly the infundibulum, akin to what has been proposed for alopecia areata.^8^ Moreover, case reports have revealed FFA with vitiligo, potentially indicating a shared pathogenic pathway. This raises the inquiry of whether topical 1.5% ruxolitinib cream, which is utilized as a treatment modality for vitiligo, would exhibit equal efficacy in treating FFA, as it is aimed at reducing underlying inflammatory processes.^9^ Additionally, by targeting underlying inflammatory pathways, patients may experience a reduction in physical symptoms such as itching, burning or pain.

Currently, the price of 1 tube of topical ruxolitinib 1.5% cream is very costly without insurance coverage.^10^ This significant cost, coupled with challenges in obtaining insurance coverage, presents a notable barrier in receiving proper treatment. Despite these obstacles, this case underscores the potential of topical ruxolitinib 1.5% cream as a valuable addition to FFA treatment when used in significant amounts. Furthermore, the lack of side effects experienced by the patient with this therapy offers possible additional benefit. We aim to highlight the potential expanded treatment modality of topical ruxolitinib 1.5% cream, offering another therapy in the care of FFA and encouraging broader insurance coverage to enhance patient access to this promising therapeutic option.

## Conflicts of interest

Dr Shapiro is a consultant for Lilly, Replicel Life Sciences, Thirty Madison, and DS Laboratories. Drs Shapiro and Lo Sicco have been investigators for Regen Lab and are investigators for Pfizer. Dr Lo Sicco is a consultant for Pfizer and Aquis. Authors Desai and Nohriahave no conflicts of interest to declare.
